# Mechanisms for Differential Protein Production in Toxin–Antitoxin Systems

**DOI:** 10.3390/toxins9070211

**Published:** 2017-07-04

**Authors:** Heather S. Deter, Roderick V. Jensen, William H. Mather, Nicholas C. Butzin

**Affiliations:** 1 Department of Physics, Virginia Polytechnic Institute and State University, Blacksburg, VA 24061-0435, USA; hdeter@vt.edu; 2 Center for Soft Matter and Biological Physics, Virginia Polytechnic Institute and State University, Blacksburg, VA 24061-0435, USA; 3 Department of Biology, Virginia Polytechnic Institute and State University, Blacksburg, VA 24061-0435, USA; rvjensen@vt.edu; 4 Quantitative Biosciences, Inc., Solana Beach, CA 92075, USA; will.mather@qbisci.com; 5 Department of Biology and Microbiology, South Dakota State University, Brookings, SD 57006, USA

**Keywords:** toxin–antitoxin systems, persister, RNA-seq, ribosome profiling, Ribo-Seq

## Abstract

Toxin–antitoxin (TA) systems are key regulators of bacterial persistence, a multidrug-tolerant state found in bacterial species that is a major contributing factor to the growing human health crisis of antibiotic resistance. Type II TA systems consist of two proteins, a toxin and an antitoxin; the toxin is neutralized when they form a complex. The ratio of antitoxin to toxin is significantly greater than 1.0 in the susceptible population (non-persister state), but this ratio is expected to become smaller during persistence. Analysis of multiple datasets (RNA-seq, ribosome profiling) and results from translation initiation rate calculators reveal multiple mechanisms that ensure a high antitoxin-to-toxin ratio in the non-persister state. The regulation mechanisms include both translational and transcriptional regulation. We classified *E. coli* type II TA systems into four distinct classes based on the mechanism of differential protein production between toxin and antitoxin. We find that the most common regulation mechanism is translational regulation. This classification scheme further refines our understanding of one of the fundamental mechanisms underlying bacterial persistence, especially regarding maintenance of the antitoxin-to-toxin ratio.

## 1. Introduction

Persistence is a metabolically inactive state that enables many bacterial species to maintain a subpopulation of cells that can survive harsh changes in the environment [1,2]. From a human health standpoint, persister cells are a growing problem since the metabolic dormancy that characterizes the persister state results in the persister population being multidrug-tolerant and a major contributing factor to ineffective antibiotic treatments. In addition, it has been suggested that persisters indirectly lead to antibiotic resistance; persister cells survive antibiotic treatment and are then able to acquire antibiotic resistant genes from their neighbors [3,4]. Investigations have revealed that a central regulator of bacterial persistence is a network of multiple toxin–antitoxin (TA) systems [5,6,7]. Evidence suggests that TA systems trigger persistence when rare events allow active toxins to accumulate and affect metabolic dormancy by slowing processes such as translation and transcription. A variety of bacterial species have TA systems, which are classified into types based on the mechanism the antitoxin uses to neutralize its cognate toxin [8,9,10,11]. Types I and III have RNA antitoxins that inhibit toxin synthesis or sequester their toxin, while types II, IV and V have protein antitoxins. Type II TA systems are the only type where the antitoxin protein directly binds to the toxin to form a protein–protein complex which sequesters the toxin and effectively neutralizes it [10]. In *Escherichia coli* alone, there at least 36 known TA systems, most of which are type two [6,12]. In this work, we were able to classify 10 out of 16 type II TA systems (there was insufficient data to classify the remaining six) found in *E. coli* K12 str. MG1655.

**Figure 1:**
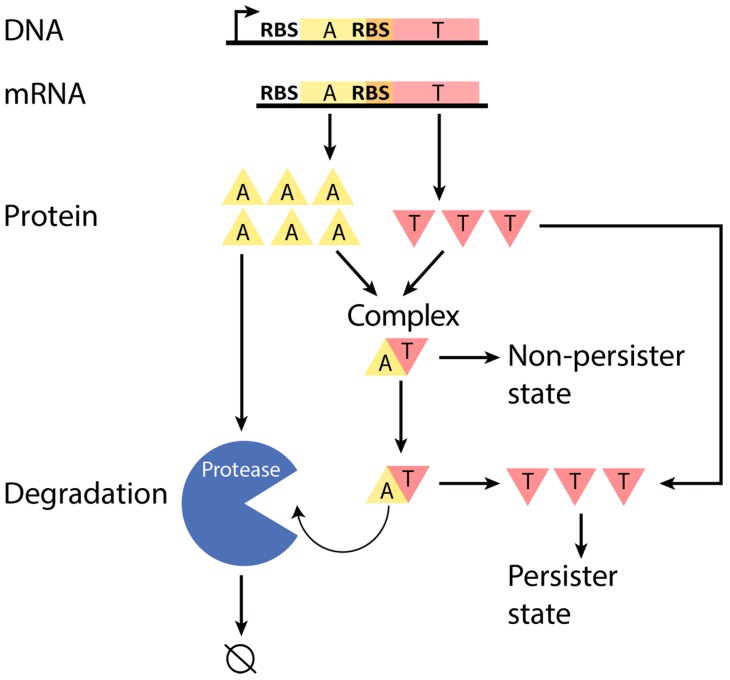
Schematic of a typical Type II toxin–antitoxin (TA) system. A TA system operon is transcribed to produce a corresponding mRNA, which is then translated to produce toxin and antitoxin proteins. With sufficient concentration of anti-toxin protein, toxin protein can be primarily neutralized in a complex, which allows the cell to maintain a non-persister state, or else the toxin can exist as a free and active protein in the cell, which leads to persistence [15]. The antitoxin-to-toxin protein ratio, which is expected to be sufficiently greater than 1.0 in the non-persister state, controls these scenarios. Antitoxin is actively degraded by proteases (at a greater rate than the toxin), which requires the excess production of antitoxin to ensure the non-persister state. T: toxin. A: antitoxin. Not to scale.

Type II TA systems are operons that encode genes for two proteins, a stable toxin and an unstable antitoxin. Since the antitoxin protein is rapidly degraded by proteases, it must be continually produced to prevent a buildup of free toxin protein and to maintain the susceptible population (non-persister state) (Figure 1) [11,13,14]. Thus, antitoxin is expected to be produced at a sufficiently higher rate than toxin in non-persister cells [8,15]. However, there is disagreement in the literature as to how this ratio is ensured across type II TA systems. It is often cited that transcriptional regulation is responsible for the higher production rate of antitoxin [8,16,17,18,19,20,21]. Research shows that the RnlAB system contains an internal promoter that is independently regulated and allows for the possibility of differential transcriptional regulation [22], but differential transcriptional regulation has not been confirmed to regulate the production ratio of antitoxin to toxin for most type II TA systems.

A model of TA systems that includes transcriptional regulation, conditional cooperativity, proposes that the antitoxin when complexed with its cognate toxin autoregulates the whole operon and could lead to some control of the antitoxin-to-toxin ratio [14,21], but this theory depends on antitoxin translation rates being higher than toxin [20]. Another model of TA systems also depends on the translation rate of antitoxin being higher than that of toxin, but does not require conditional cooperativity [23]. Many other operons are known to use translational regulation (different translation rates) to maintain differential protein production within an operon, including the ATPase operon [24,25]. As we will support, many type II TA systems use translational regulation, and transcriptional regulation is not the only mechanism that can explain the higher production rate of the antitoxin protein.

This study examines the possibilities of both transcriptional and translational regulation using bioinformatics approaches that combine diverse datasets and analyses. We analyzed type II TA systems in *E. coli* K12 str. MG1655 using RNA sequencing (RNA-seq) data from multiple studies and growth conditions, data from ribosome profiling analysis (Ribo-Seq), identified promoters and terminators with experimental data and predictions annotated on EcoCyc [26], and calculated translation initiation rates with prediction algorithms (TIR calculators). By combining these analyses, we classified *E. coli* type II TA systems into four classes based on the mechanisms of differential protein production. This classification scheme further refines our understanding of how TA systems maintain differential antitoxin-to-toxin expression and one of the fundamental mechanisms underlying bacterial persistence.

A major result of our investigation is that differential transcriptional regulation of antitoxin and toxin expression is unlikely in many type II TA systems. The key pieces of evidence are a less than two-fold difference of antitoxin-to-toxin mRNA for several systems, and a lack of obvious internal promoters or terminators within the operon sequence that could explain excess antitoxin mRNA. We predict that TA systems with less than a two-fold difference in mRNA expression leverage translational regulation to maintain higher production rates of antitoxin. Our results extend the pattern found for many operonic genes that use translational regulation to maintain differential protein production from a single transcript [24,25,27].

## 2. Results and Discussion

We analyzed 10 different type II TA systems in *E. coli* (Table 1) using data informing their operon organization, mRNA concentration (RNA-seq), protein synthesis rates (Ribo-Seq), and predicted translation initiation rates (TIR’s). Six type II TA systems lacking substantial RNA-seq data were not included. Understanding of the operon organization and mRNA products for each TA system led to a compelling classification scheme that includes at least four major classes. We find that these classes of TA systems all have mechanisms in place to ensure sufficient production of antitoxin protein relative to toxin protein, though the details vary from class to class. In this section, we present the major findings of our study. We first present the details concerning our TA system classification scheme; then we analyze trends in associated quantitative data. We finally discuss our interpretation of these results in terms of the four identified classes of TA systems.

### 2.1. Classification Scheme for Type II Toxin–Antitoxin Systems Based on DNA Sequence and mRNA Products

A survey of 10 TA systems (Table 1) using the online database EcoCyc [26], revealed consistent patterns based on operon organization, which refers to the order of the genes (whether the toxin is upstream at the 5′ end or downstream at the 3′ end of the operon), and whether there is an additional promoter or transcriptional termination mechanism that can produce multiple distinct mRNA products.

**Figure 2:**
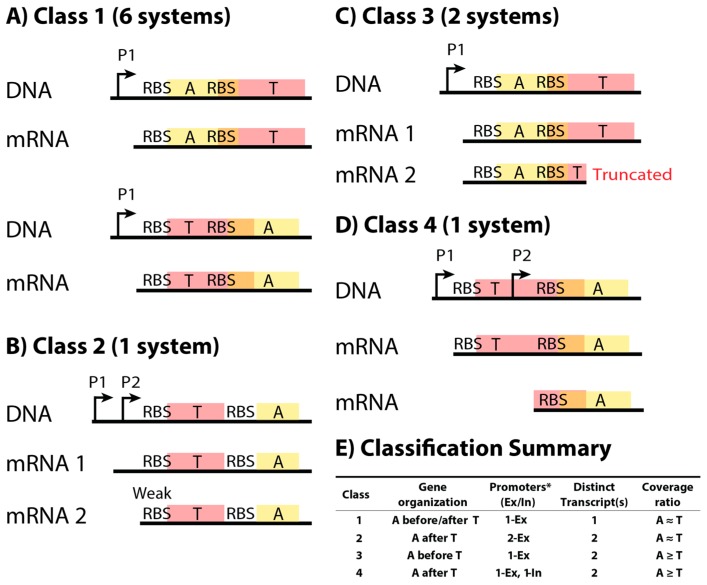
TA (toxin–antitoxin) systems were classified into four different classes based on DNA sequence and measured mRNA products. (**A**–**D**) Representative wire diagrams of the operon organization and mRNA products of each class (not to scale). (**A**) Class 1 TA systems (FicAT, MazEF, MqsAR, PrlF-YhaV, RelBE, YefM-YoeB), the most abundant class in our study, have a single transcript for the operon and should rely on translational regulation to ensure higher antitoxin production relative to toxin production; (**B**) Class 2 is characterized by a second promoter that produces a slightly shorter transcript, which is predicted by our work to have a lower toxin translation rate than the transcript of the first promoter. The one example (HicAB) available has non-overlapping coding regions with the toxin upstream from the antitoxin; (**C**) Class 3 TA systems (DinJ-YafQ, YafNO) have a truncated transcript in addition to the whole transcript for the operon, due to some unknown mechanism (perhaps a terminator or post-transcriptional degradation); (**D**) Class 4 TA systems (RnlAB) produce two transcripts: a transcript of the whole operon (both toxin and antitoxin mRNA), and a transcript that can only be translated to antitoxin; (**E**) A summary of the classification of TA systems in this study. For the promoter column, the number indicates the number of promoters that are located upstream the coding regions (external, Ex), or within the coding regions (internal, In). Each class has a different A-to-T RNA ratio (see Table 2) based on analysis of 13 different RNA-seq datasets from a variety of conditions, such as growth in rich and minimal media, and cell densities (Table S1). * Some genes may have additional promoters, but they did not affect the ratio of mRNA expression. P: Promoter. RBS: Ribosome Binding Site. A: Antitoxin. T: Toxin.

Further analysis of our representative set of TA systems led to one major class and three other classes (Figure 2). The major class (Class 1: FicAT, MazEF, MqsAR, PrlF-YhaV, RelBE, YefM-YoeB) found in our study includes TA systems that produce a single transcript, which results in the condition A ≈ T for mRNA (the concentration of antitoxin coding region is approximately equal to the concentration of toxin coding region). Because these systems are apparently not differentially regulated at the transcriptional level, we predict that different translation rates of the two proteins are responsible for higher antitoxin protein production than toxin. These predictions are confirmed later in this article. The three other classes (Class 2: HicAB; Class 3: DinJ-YafQ, YafNO; and Class 4: RnlAB) are inspired by a few systems that have similar gene organization to Class 1, but other features deviate from Class 1 that allow for transcriptional or post-transcriptional regulation of the antitoxin to toxin protein production ratio. Interestingly, we find the ribosome-binding site (RBS) of the downstream gene is embedded in the upstream gene for nine out of 10 TA systems (only HicAB in Class 2 does not).

Many of these classes were identified by examining mRNA expression from RNA-seq data in combination with promoter identification (see Methods). Class 2 is similar to Class 1, but an additional external promoter (located before the coding regions) is near the toxin RBS. Transcription from this promoter results in a truncated mRNA that has a weakened RBS for toxin translation. Thus, antitoxin is produced at a greater rate than toxin [38]. Class 3 likely has a truncated mRNA according to our analysis of RNA-seq data, but due to the gene organization of this class, it probably has either an early transcriptional terminator or post-transcriptional mRNA degradation to truncate the toxin-coding region. Class 4 has an internal promoter that produces excess antitoxin mRNA. Classes 3 and 4 rely on increased abundance of functional antitoxin-coding mRNA relative to toxin. Thus, a higher TIR for antitoxin relative to toxin is perhaps then not as necessary, in contrast to Class 1.

### 2.2. Antitoxin and Toxin mRNA Coverage by RNA-seq

Our classification scheme (Figure 2) was supported using a diverse-data analysis approach. As part of this analysis, we quantitatively estimated the mRNA abundance for antitoxin and toxin coding regions using publicly available RNA-seq data. A total of 13 different whole transcriptome *E. coli* K12 str. MG1655 RNA-seq datasets were derived from two different studies with a total of six different experimental conditions that include different growth densities and media (Table S1).

**Figure 3:**
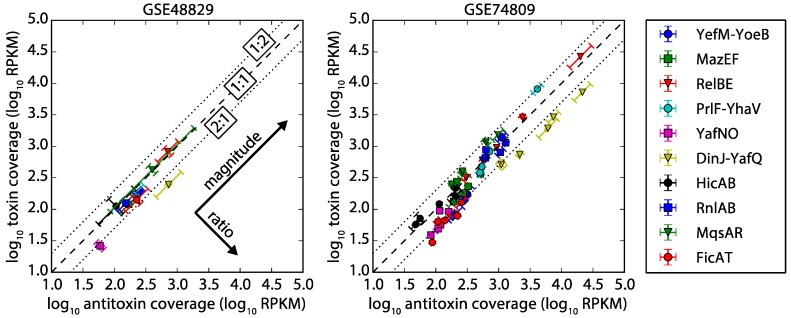
RNA-seq coverage of antitoxin vs. toxin open reading frames. Left: dataset GSE48829 [26] triplicate biological replicates sampled during exponential growth in minimal media. Right: dataset GSE74809 [27] duplicate biological replicates sampled from five different stages of growth in M9 (glucose) media. Both plot the quantitative analysis of sequence coverage of antitoxin and toxin (see Methods) on a common axis for a variety of TA systems. Most TA systems in the conditions considered have less than a two-fold difference in coverage (1:1 coverage is indicated by a dashed line) between antitoxin-to-toxin mRNA, suggesting expression of the mRNA as a single transcript. TA systems that fall within the dotted lines had a 1:2 to 2:1 ratio of antitoxin to toxin coverage. TA systems were not included if either toxin or antitoxin had an average of less than one read per base for more than half of the datasets (the minimum read count for a gene is 168 reads). The error was calculated in two different directions (ratio and magnitude, see Methods), and error bars are aligned to these primary directions to illustrate the low error of the ratio. The individual replicates had similar groupings (Figure S2). Our results are also supported by a global error analysis (Figure S3), which shows a typically small error for the replicates. Units of coverage are Reads Per Kilobase Million (RPKM).

RNA-seq analysis shows that the sequencing coverage (number of reads aligned to the gene normalized by dividing the length of the gene) for seven TA systems had less than a two-fold difference in expression at the mRNA level for a variety of conditions (Table 2, Figure 3), as anticipated for Class 1 and Class 2 TA systems. The two TA systems in Class 3 (DinJ-YafQ, YafNO) consistently had more antitoxin mRNA than toxin, which is also as anticipated, though the two-fold difference is modest. Our one example of a Class 4 system (RnlAB) also had less than a two-fold difference between toxin and antitoxin mRNA expression, but the coverage of functional antitoxin mRNA is higher than functional toxin mRNA (Figure S1).

### 2.3. Protein Synthesis Rates Determined by Ribo-Seq

**Figure 4:**
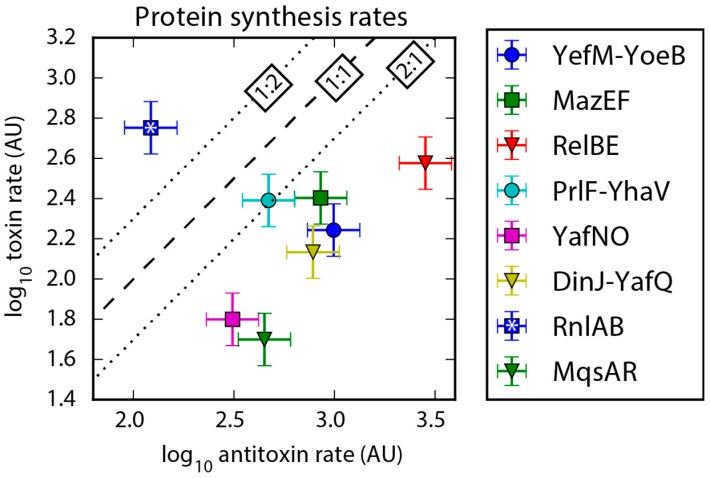
Protein synthesis rates plotted for each TA system based on Ribo-Seq data. A systematic bias towards higher antitoxin protein synthesis rate relative to toxin is evident in all but one case, and we explain the one outlier RnlAB in Section 2.5. The dashed line represents a 1:1 ratio of antitoxin-to-toxin, and the dotted lines mark a two-fold difference. TA systems with values of low confidence (less than 128 reads) in protein synthesis data [39] were not included in the figure. Error bars assume a 30% error, as estimated in Li et al. 2014 [39]. Arbitrary units: AU.

Sequencing coverage of mRNA was suggestive, in that most TA systems had less than a two-fold difference in coverage of antitoxin and toxin. To test our classification of type II TA systems into four classes based on gene organization and RNA-seq data, we analyzed protein synthesis rates from publicly available Ribo-Seq data. Indeed, the Ribo-Seq data supports our classification. Protein synthesis rates were determined using the Li et al. (2014) open source database that gives protein synthesis rates for *E. coli* based on Ribo-Seq data [39] (See Methods). Protein synthesis rates were calculated from Ribo-Seq based on the coverage (counts normalized by gene length) of the ribosomally protected mRNA present after the mRNA has been extracted and treated with RNase [40]. From this analysis, seven out of eight of the TA systems with sufficient coverage (HicAB and FicAT had low confidence, less than 128 mapped reads) had a higher protein synthesis rate for the antitoxin than the toxin (Figure 4), as anticipated. We hypothesize that the systems with less than a two-fold difference in toxin and antitoxin mRNA expression likely use translational regulation to maintain the higher antitoxin production rate. The major exception we found was the synthesis ratio for the RnlAB TA system (Class 4), which we hypothesize may be due to third protein that interacts with this system (see comments in Section 2.5).

### 2.4. Analysis of Differential Protein Expression Using Translation Initiation Calculators (TIRs)

An independent theoretical investigation of TIRs for the 10 TA systems was done to assess the robustness of our findings based on Ribo-Seq data. We predicted TIRs using three experimentally-conditioned TIR calculators, of which two are based on detailed thermodynamic models (the RBS Calculator [41,42] and the UTR Designer [43]), and a third is based on a log-linear regression of *E. coli* gene expression (Barrick Calculator [44]). The results from the TIR calculators vary greatly due to the inherent differences between the calculation methods, but for six of the seven systems that have less than a two-fold difference in mRNA expression, at least two out of three calculation methods qualitatively agree that for each system the antitoxin TIR is higher than toxin (Table 3 and Table S2). In all cases, the calculators support our classification of the TA systems and provide a method independent of Ribo-Seq.

### 2.5. Summary and Discussion of Major Trends and Exceptions for TA System Classes

Our picture from a diverse-data analysis of 10 TA systems has thus led to the following key interpretations of their behavior:

Class 1 is the most abundant class with six TA systems that use translational regulation to maintain a high antitoxin-to-toxin protein synthesis rate, as seen in the Ribo-Seq analysis (Table 2, Figure 4). The TA systems in this class have less than a two-fold difference in mRNA expression (Table 2, Figure 3) and likely use translation regulation to maintain the antitoxin-to-toxin ratio. The RBS site of the downstream gene in these systems overlaps with the upstream gene’s coding region, and they are not dependent upon the order of those genes to maintain the antitoxin-to-toxin production ratio (Figure 2A). Included in this class is RelBE, which previous researchers have shown to use translational regulation to maintain a high antitoxin-to-toxin ratio [16]. The only TA system in this study to clearly use translational regulation that is not in Class 1 is HicAB, which we have placed in Class 2.

The protein synthesis rates of HicAB could not be determined by the Li et al. (2014) data due to low expression of this system, but our RNA-seq analysis shows that the HicAB system has less than a two-fold difference in mRNA expression, like Class 1. Unlike Class 1, HicAB does not have overlapping genes and the antitoxin is located downstream of the toxin (Figure 2B). Our first analysis showed that translational regulation is unlikely because two out of three TIR calculators predict that the antitoxin TIR is lower than the toxin. Interestingly, a recent study showed that HicAB combines transcriptional and translational regulation by using two different promoters at the beginning of the operon to produce two different transcripts with different toxin translation rates [38]. When using the transcription start site of the second promoter, the results of the RBS Calculator change to predict the antitoxin TIR to be higher than the toxin. This entails in two of the three TIR calculators predicting antitoxin TIR is higher than toxin (Table S2), supporting that this system uses translational regulation and supporting the effectiveness of the TIR calculators when using a consensus of two out of three.

Class 3 contains the remaining two systems that had higher antitoxin protein synthesis rates (Figure 4), but these systems also have two-fold higher antitoxin mRNA expression than toxin (Table 2, Figure 3). The organization of these systems has the antitoxin upstream of the toxin with the RBS of the toxin overlapping the end of the antitoxin-coding region (Figure 2C). While the difference in mRNA would indicate that these systems are transcriptionally regulated, the regulation mechanism apparently does not use a promoter; the antitoxin location upstream of the toxin would result in an additional promoter for the antitoxin reading through the toxin as well. We hypothesize that the lower toxin mRNA level is a result of a truncated mRNA due to an unidentified transcriptional terminator or RNA degradation specific to the toxin mRNA sequence. Several type II toxins are endoribonucleases (including those in Class 3) and they could possibly degrade their own message with a bias toward toxin mRNA degradation (Table 1). Regardless of the mechanism, Class 3 TA systems likely use differential mRNA expression to regulate antitoxin-to-toxin ratios either transcriptionally with terminators or post-transcriptionally through RNA degradation.

Class 4 contains RnlAB, the only TA system that does not have a higher antitoxin protein synthesis rate than toxin (Figure 4). The gene organization of this system has the antitoxin downstream of the toxin with the antitoxin RBS overlapping the end of the toxin-coding region (Figure 2D). This organization means that the RnlAB system can use transcriptional regulation of an internal promoter to express antitoxin mRNA higher than toxin, and analysis of the RNA-seq data supports the presence of an internal promoter at approximately 280 nucleotides upstream from the antitoxin (Figure S1). The RNA-seq analysis is supported by a previous study on the regulation of RnlAB, which experimentally determined an internal promoter to be near this location. The same study establishes that the two promoters are independent and transcriptionally regulated, which would allow for upregulation of antitoxin expression [22]. The internal promoter in this system provides a possible mechanism to upregulate antitoxin production, but the protein synthesis rates indicate that toxin production is higher than antitoxin production.

The toxin homologs RnlA and LsoA (not found in the *E. coli* strain used in this study) are unlike other type II TA systems because the protein structure of these toxins is different from established structures of others. Additionally, a third protein interacts with the RnlAB system, RNase HI. Recent studies have shown that RNase HI acts as a corepressor of the toxin RnlA in the presence of its antitoxin RnlB [45]. However, when antitoxin is not present in the system (in the absence of RnlB), RNase HI stimulates RnlA activity [46]. This system also acts as an anti-phage response, because during infection RnlA degrades the infecting phage’s mRNA and RnlB concentration decreases allowing RNase HI to activate RnlA and strengthen its activity [45,46]. The third component of the RnlAB system changes the dynamics and is likely the reason that the ratio of antitoxin protein synthesis rate to the toxin is lower than expected.

### 2.6. Incorporation of Our Results into Current Models

Currently, many studies on regulation of TA systems focus on conditional cooperativity, which depends on transcriptional regulation by TA autoregulation complexes. Numerous studies on TA autoregulation complexes focus on RelBE, a Class 1 system. The RelBE system is known to produce the antitoxin (RelB) approximately 10-fold faster than the toxin (RelE) [16], which is further supported by the protein synthesis rate data, which predicts RelB to be produced 7.5 times greater than RelE [39]. Studies on RelBE show that DNA binding of the RelB_2_E complex is stronger than the RelB_2_ alone [47,48], and studies suggest that the stronger autoregulation activity of the complex is important to the control of the antitoxin-to-toxin ratio [20,47]. However, in these models, the translation rate of antitoxin being higher than that of the toxin is critical to prevent a constant overabundance of free toxin, and therefore the system is still dependent on translational regulation [16,20]. In contrast to the TA autoregulation complex of the RelBE system, the DinJ-YafQ complex does not have increased DNA binding affinity when compared to DNA binding affinity of the antitoxin (DinJ) alone [49]. Interestingly, the DinJ-YafQ system was placed into Class 3, which our results suggest relies on increased abundance of antitoxin mRNA rather than translation rates. Further studies are needed to fully understand the variety of regulation mechanisms that play a role in the maintenance and control of the antitoxin-to-toxin ratio.

## 3. Conclusions

Both translational and transcriptional regulation play important roles in the maintenance of the production ratio of antitoxin-to-toxin in type II TA systems in the non-persister state. Analysis of RNA-seq data reveals that most TA systems have less than a two-fold difference between antitoxin mRNA and toxin under the conditions studied in these datasets (see Table S2). The antitoxin-to-toxin production ratio in Class 1 TA systems, which represent the most abundant class in our study, is not differentially regulated transcriptionally or post-transcriptionally, but it is regulated at the translational level. Class 2 uses a combination of both transcriptional and translational regulation to ensure a higher rate of antitoxin production. Class 3 is likely not regulated translationally but by some mechanism that results in a truncated mRNA and overall greater expression of antitoxin mRNA than toxin. Class 4 contains RnlAB, which is regulated by internal promoters, but the interaction of RNase HI with RnlAB lessens its dependency on a higher production rate of antitoxin.

Our classification of TA systems emphasizes their diversity with respect to gene organization and regulation mechanisms, while other classification systems are based on protein structures and functions [10,13]. The diversity of regulation mechanisms explains the disagreement in the literature as to whether TA systems use transcriptional regulation or some other form of regulation to maintain an antitoxin production rate higher than the cognate toxin. Various studies have focused on specific TA systems and their conclusions have been applied to type II TA systems as a group, but our results emphasize the diversity found within these systems. Further applications of the methods used in this study would be to classify and expand the knowledge of TA systems in other organisms and other differentially expressed operons in bacteria. Using bioinformatics methods alone, the methods in this study can be applied to classify other type II TA systems. Classification of different type II TA systems into these classes should allow future researchers to predict regulation (transcriptional, post-transcriptional, or translational) without the expense and time of RNA-seq and Ribo-Seq experiments.

## 4. Materials and Methods

### 4.1. DNA Sequence and mRNA Sequence Analysis

The annotated *E. coli* K12 str. MG1655 reference sequence NC_000913.3 was used for all gene sequences in this study.

### 4.2. RNA-seq Analysis

Selected RNA-seq datasets (Table S1) from GEO NCBI (accession numbers GSE48829 and GSE74809) [50,51] were aligned to the *E. coli* K12 str. MG1655 reference sequence NC_000913.3 using Geneious v. 10.0.9 [52]. Gene expression was analyzed in Geneious using the calculate expression tool to generate a list of reads mapped to each gene. Coverage for any specific antitoxin or toxin was calculated in Reads Per Kilobase Million (RPKM). Defining n as the raw number of reads mapped to the coding sequence (CDS) (with partial reads, reads mapping to more than one region of the genome, counted as 0.5 reads), T as the total number of mapped reads for a particular RNA-seq dataset, and L as the number of bases in the CDS, then RPKM = $10^{9}\;n/LT$. Results were exported to a CSV file, which was then processed by custom Python scripts using the NumPy library [53].

The ratio of antitoxin to toxin was calculated for each TA system for all 13 datasets, and the median of those 13 ratios was used to determine the A/T ratio. The standard deviations were calculated using the 13 ratios from the datasets (Table 1).

Error estimates for gene coverage were computed as follows. Each RNA-seq dataset contained biological replicates, and we analyzed error by comparing gene expression between replicates (see Figure S2 for an illustration of replicates). Dataset GSE48829 contained triplicate data for one experimental condition, while dataset GSE74809 contained duplicate data for five experimental conditions. For each dataset separately, we ran an error analysis of the log-error (standard error of logarithmic quantities) in two major directions, ratio and magnitude. We choose log-error since this is the most natural representation of the error for features plotted in log-space, as with Figure 3. If $c_{A}$ and $c_{T}$ are gene coverages for antitoxin and toxin, respectively, then the error of the log-ratio is the standard error for the quantity $\ln c_{A}-\ln c_{T}=\ln\left(c_{A}/c_{T}\right)$. Since $\ln\left(c_{A}/c_{T}\right)\approx \left(c_{A}/c_{T}\right)-1$ when $c_{A}$ and $c_{T}$ are close in value, this error also approximates the error of the ratio (the constant -1 does not contribute to the standard error). This error is plotted as error bars along the ratio direction in Figure 3 after scaling by the factor ln(10) to plot in logarithm base 10 space. A complementary error estimate is that of the log-magnitude, which we define as the standard error of the quantity $\ln c_{A}+\ln c_{T}=\ln\left(c_{A}\cdot c_{T}\right)$, and which we plot as error bars along the magnitude direction in Figure 3 after scaling by ln(10). Notice that the log-ratio error is zero for quantities with zero uncertainty in the ratio $\left(c_{A}/c_{T}\right)$, even though the log-magnitude error may be significant.

### 4.3. Protein Synthesis Rates Based on Ribosome Profiling (Ribo-Seq)

We used protein synthesis rates for *E. coli* calculated by Li et al. 2014 [39] from their Ribo-Seq data according to their open source database. Genes with less than 128 mapped reads were given values of low confidence in this database.

### 4.4. Translation Initation Rate (TIR) Calculators

TIRs were predicted using three different translation rate initiation (TIR) calculators with the reference sequence, NC_000913.3. The reverse engineering feature of the Ribosome Binding Site Calculator v2.0 was used [41,42]. Any difference in sequence length using the RBS Calculator v2.0 changed the translation initiation rates because this software accounts for secondary structure. Therefore, the input sequence we used included the entire 5′ UTR region as determined by the transcription start site annotated in the EcoCyc database [26] to the end of the TA system. The reverse engineering feature of the UTR Designer was used with the required input of −25 to +35 from the start codon for each gene [43]. The third method analyzed the sequence −11 to +1 from the start codon using an equation based on empirical data developed by Barrick et al. in their study of *E. coli* ribosome binding sites (Barrick Calculator) [44]. We used the agreement of two out of three calculators to determine whether antitoxin TIR is predicted to be higher than toxin.

## Figures and Tables

**Table 1:** Type II TA systems examined in this study, ordered alphabetically. Ten out of sixteen type II TA systems (there was insufficient data to classify the remaining six) in *E. coli* were considered. The toxin is underlined.

TA System	Toxin Function	Toxin Family
DinJ-YafQ	Endoribonuclease that act 5′ to adenine between the codon second and third nucleotides [28]	RelE [13]
FicAT	Mediates post-translational protein modification [29]	Unknown
HicAB	mRNase [30]	Unknown [31]
MazEF	mRNA interferase that cleaves mRNA at ACA sites [32]	CcdB/MazF [13]
MqsAR	Ribosome-independent RNase [33]	RelE [31]
PrlF-YhaV	Ribonuclease [34]	RelE [31]
RelBE	mRNA interferase that cleaves mRNA in the ribosome A site [35]	RelE [13]
RnlAB	RNase [22]	Unknown [31]
YafNO	Ribosome-dependent mRNA interferase [36]	YafO [13]
YefM-YoeB	Ribosome-dependent mRNase [37]	RelE [13]

**Table 2:** Median of the ratios for antitoxin-to-toxin coverage based on RNA-seq. The median (n = 13) of the antitoxin to toxin coverage ratios calculated for each of the 13 datasets (see Methods). All TA systems exhibited nearly equal (less than a two-fold difference) or higher antitoxin mRNA abundance. TA systems are ordered by their class. The toxin is underlined. * Although near the two-fold cut off, these systems are placed in Class 1 from additional analysis (Table 3). ** A truncated mRNA results in differing levels of functional mRNA (Figure S1).

TA System	A/T	St. Dev	Class
FicAT *	1.99	0.53	1
YefM-YoeB *	1.89	0.42	1
**MazEF**	1.42	0.25	1
**PrlF-YhaV**	1.11	0.27	1
**MqsAR**	0.69	0.16	1
**RelBE**	0.95	0.10	1
**HicAB**	0.93	0.20	2
**DinJ-YafQ**	2.89	0.35	3
**YafNO**	2.08	0.39	3
RnlAB **	1.01	0.25	4

**Table 3:** Classification of each TA system. Based on the classification scheme outlined in Figure 2, we used qualitative conclusions from our analysis to classify 11 different TA systems in *E. coli*. Data from RNA-seq was used to determine mRNA ratio. The toxin is underlined.

TA System	Class	mRNA Ratio	RBS Calculator	UTR Designer	Barrick Calculator	Synthesis Rates
FicAT	1	<2 *	+	+	+	LC
MazEF	1	<2	+	+	+	+
MqsAR	1	<2	−	+	+	+
PrlF-YhaV	1	<2	+	+	+	+
RelBE	1	<2	+	+	+	+
YefM-YoeB	1	<2 *	+	+	+	+
HicAB P1	2	<2	−	−	+	LC
HicAB P2			+	−	+	
DinJ-YafQ	3	>2	+	−	−	+
YafNO	3	>2	+	−	−	+
RnlAB	4	<2 **	+	+	−	−

+: antitoxin is higher than toxin. −: toxin is higher than antitoxin. LC: low confidence; * mRNA ratio ranges broadly (sometimes > 2) but calculator results place these systems in Class 1; ** A truncated mRNA results in differing levels of functional mRNA (Figure S1).

## Supplementary Materials

The following are available online at www.mdpi.com/2072-6651/9/7/211/s1, Figure S1: RNA-seq reads from experiment SRX1424838 (GSE74809) mapped to *mazEF*, and *rnl*AB; Figure S2: Illustration of RNA-seq coverage for all replicates and conditions used; Figure S3: Global error analysis of RNA-seq coverage; Table S1: Selected RNA-seq experiment numbers and conditions for GSE48829 (grey background) and GSE74809; Table S2: Ratios of the calculated antitoxin to toxin translation initiation rates.
