# Supplementary material for: Mechanisms for Differential Protein Production in Toxin–Antitoxin Systems
Source: Toxins (Basel). 2017 Jul 4;9(7):211. doi: 10.3390/toxins9070211 (PMC5535158; doi:10.3390/toxins9070211)
Supplement: Supplementary file 1 [file toxins-09-00211-s001.pdf]

Supplementary Materials: Mechanisms for Differential Protein Production in Toxin–Antitoxin Systems

Heather S. Deter, Roderick V. Jensen , William H. Mather and Nicholas C. Butzin

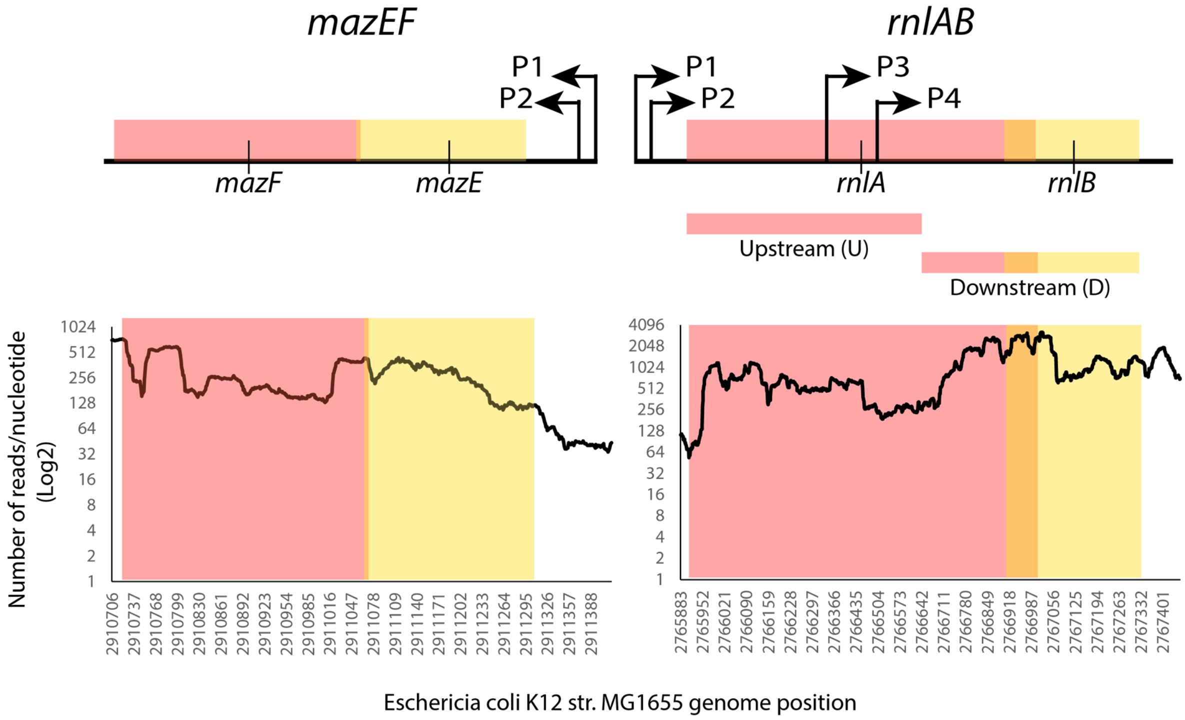

Gene	Mean coverage
mazE	234
mazF	281
mazEF	261

Coverage ratios *mazEF*	
E/ F	0.8
E/ EF	0.9
F/ EF	1.1

Gene	Mean coverage
rnlA	915
rnlB	1302
rnlAB	1015
rnlA (U)	708
rnlAB (D)	1554

Coverage ratios *rnlAB*	
B/A	1.4
B/AB	1.3
A/AB	0.9
D/U	2.2
D/AB	1.5
U/AB	0.7

Figure S1. RNA-seq coverage from experiment SRX1424838 (GSE74809, [1]) mapped to *mazEF* (Class 1), and *rnl*AB (Class 4). The *mazEF* genes do not have greater than a twofold difference in coverage (number of RNA-seq reads mapped to the genome) or an internal promoter according to the EcoCyc database [2]. The coverage of *rnlAB* shows an increase of transcription at the transcriptional start site for the internal promoter (P4) (indicated as a dashed line) approximately 280 nt upstream from the antitoxin start codon [3]. Comparison of the coverage between toxin (*rnlA*) and antitoxin (*rnlB*) using number of reads mapped to each gene would misrepresent the ratio of mRNA due to the transcriptional start site located within the *rnlB* gene. The coverage before the internal promoter is twofold lower than the coverage after the internal promoter, indicating that functional toxin mRNA is two-fold less than functional antitoxin mRNA. **Top:** Wire diagram of the indicated genes above the corresponding line graph of the coverage on a $\log_2$ scale versus nucleotide position in the genome. P: promoter **B)** Table indicating the mean coverage and coverage ratios for a given gene or region.

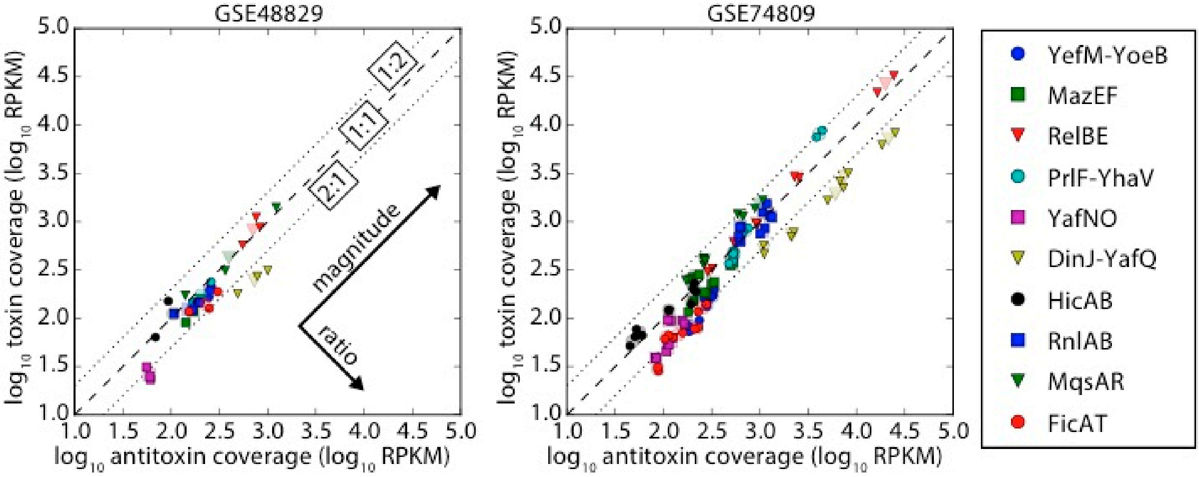

Figure S2. Biological replicate values for antitoxin and toxin coverage across RNA-seq datasets. Shown is a comparison of coverage for biological replicates from the datasets GSE48829 [4] (left) and GSE74809 [1] (right), the former of which contains triplicate data in one growth condition, and the latter of which contains duplicate data across five growth conditions. Replicates are shown as small symbols, while the mean of their $\log_{10}$ coverage is shown as a corresponding larger transparent symbol. The dashed line represents antitoxin to toxin coverage ratio 1:1 (equal coverage), while the dotted lines represent antitoxin to toxin coverage ratios equal to 1:2 and 2:1. Units of coverage are Reads Per Kilobase Million (RPKM), and the major directions of ratio and magnitude are also included (see Methods).

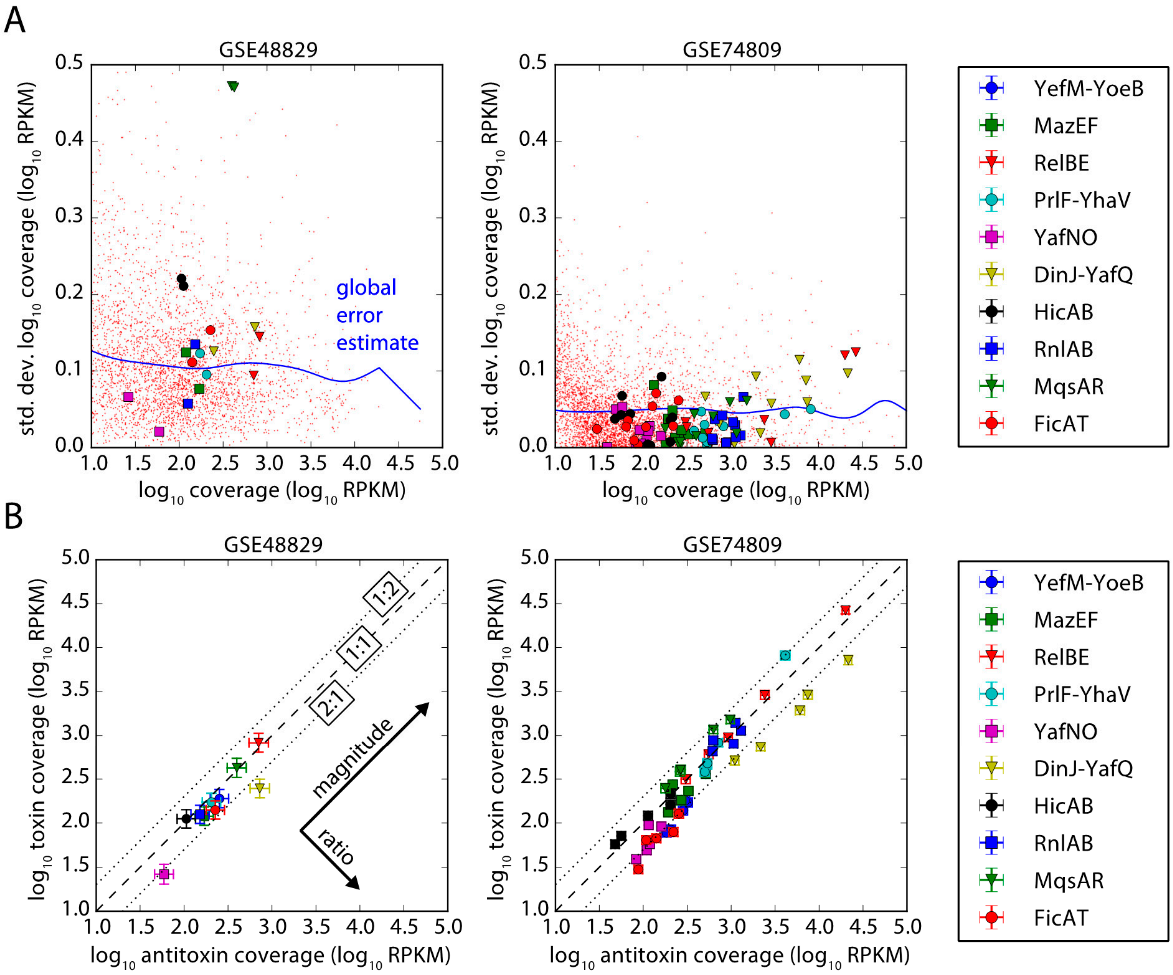

Figure S3. Representative error estimates for RNA-seq datasets. (**A**) Biological replicates in the datasets GSE48829 [4] (left) and GSE74809 [1] (right) were used to estimate the standard error of the log-coverage (natural logarithm of the coverage) for each gene in the dataset (red dots). This logarithmic error measurement is natural for data represented in log-log coordinates. Antitoxin and toxin genes belonging to the TA systems listed in the legend are represented using their own symbols. A smooth global error estimate is plotted as a blue line. This global error estimate $\langle\sigma\rangle_i$ for a gene with index i is derived from the formula $\langle\sigma\rangle_i = \sum_j \sigma_j \rho_{ji}$ (summation over all indices j), where ρ_{ji} is a normalized weighting factor proportional to $\exp\left(-2\left(x_i - x_j\right)^2\right)$, with x_i the log-coverage for a gene with index i. (**B**) The mean log-coverage for each condition from panel A are plotted, with error bars corresponding to their global error estimates.

Table 1. Selected RNA-seq experiment numbers and conditions for GSE48829 (grey background) and GSE74809.

Experiment	Media	Growth phase
**SRX322083**	Minimal media	Exponential
**SRX322084**	Minimal media	Exponential
**SRX322085**	Minimal media	Exponential
**SRX1424798**	M9 (glucose)	Early Exponential
**SRX1424799**	M9 (glucose)	Early Exponential
**SRX1424808**	M9 (glucose)	Mid-Exponential
**SRX1424809**	M9 (glucose)	Mid-Exponential
**SRX1424818**	M9 (glucose)	Transition to Stationary
**SRX1424819**	M9 (glucose)	Transition to Stationary
**SRX1424828**	M9 (glucose)	Stationary
**SRX1424829**	M9 (glucose)	Stationary
**SRX1424838**	M9 (glucose)	Late Stationary
**SRX1424839**	M9 (glucose)	Late Stationary

Table 2. Ratios of the calculated antitoxin to toxin translation initiation rates. All translation initiation rates (TIR) were calculated using translation rate calculators, as outlined in the main text (see Methods). TIR is in arbitrary units (AU). The toxin is underlined.

TA System	RBS Calculator	UTR Designer	Barrick Calculator
**MazE<u>F</u>**	8.93	1.94	6.05
**PrlF-<u>YhaV</u>**	262	4.97	1.39
**RelB<u>E</u>**	17.8	2.84	1.45
**MqsA<u>R</u>**	0.01	1.34	15.1
**YefM-<u>YoeB</u>**	1.48	3.83	72.9
**DinJ-<u>YafQ</u>**	1.36	0.46	0.23
**YafN<u>O</u>**	10.4	0.97	0.32
**Rnl<u>A</u>B**	119	NA	0.15
**FicA<u>T</u>**	31.4	18.9	1.20
**Hic<u>A</u>B P1**	0.10	0.18	7.61
**Hic<u>A</u>B P2**	8.93	1.94	6.05

N/A: Not available because the UTR Designer calculator cannot calculate with a TTG start codon.

References

1. Lal, A.; Krishna, S.; Seshasayee, A.S.N. Regulation of global transcription in *E. coli* by Rsd and 6S RNA. **2016,** doi: https://doi.org/10.1101/058339.
2. Karp, P.D.; Weaver, D.; Paley, S.; Fulcher, C.; Kubo, A.; Kothari, A.; Krummenacker, M.; Subhraveti, P.; Weerasinghe, D.; Gama-Castro, S., *et al.* The EcoCyc Database. *EcoSal Plus* **2014**, *6*.
3. Otsuka, Y.; Miki, K.; Koga, M.; Katayama, N.; Morimoto, W.; Takahashi, Y.; Yonesaki, T. IscR regulates RNase LS activity by repressing *rnlA* transcription. *Genetics* **2010**, *185*, 823–830.
4. Meysman, P.; Sonego, P.; Bianco, L.; Fu, Q.; Ledezma-Tejeida, D.; Gama-Castro, S.; Liebens, V.; Michiels, J.; Laukens, K.; Marchal, K., *et al.* COLOMBOS v2.0: an ever expanding collection of bacterial expression compendia. *Nucleic Acids Res* **2014**, *42*, D649–653.
